# Mapping the proteome landscape in *Euglena gracilis*: global, phospho, and ubiquitin proteomes under anaerobic response

**DOI:** 10.1080/15592324.2026.2720310

**Published:** 2026-10-08

**Authors:** Yosua, Shun Tamaki, Kohei Nishino, Akihiro Ezoe, Anzu Minami, June-Sik Kim, Ade Rizqi Ridwan Firdaus, Toto Subroto, Hidetaka Kosako, Keiichi Mochida

**Affiliations:** a Bioproductivity Informatics Research Team, RIKEN Center for Sustainable Resource Science, Yokohama, Japan; b Kihara Institute for Biological Research, Yokohama City University, Yokohama, Japan; c Department of Biological Science, Faculty of Science and Engineering, Yasuda Women's University, Hiroshima, Japan; d Division of Cell Signaling, Institute of Advanced Medical Sciences, Tokushima University, Tokushima, Japan; e Institute of Plant Science and Resources, Okayama University, Kurashiki, Japan; f Department of Chemistry, Faculty of Mathematics and Natural Sciences, Universitas Padjadjaran, Jatinangor, West Java, Indonesia; g Research Center for Molecular Biotechnology and Bioinformatics, Universitas Padjadjaran, Bandung, West Java, Indonesia; h RIKEN Baton Zone Program, RIKEN Cluster for Science, Technology and Innovative Hub, Yokohama, Japan; i School of Information and Data Sciences, Nagasaki University, Nagasaki, Japan

**Keywords:** *Euglena gracilis*, proteome, phosphorylation, ubiquitination

## Abstract

The photosynthetic flagellate *Euglena gracilis* (Euglena) exhibits a remarkable metabolic flexibility, enabling the dynamic coordination of energy production and resource partitioning during anaerobic adaptation. While the wax ester synthesis under hypoxia is well-documented, the regulatory interplay between protein abundance and post-translational modifications (PTMs) remains unexplored. A definitive understanding of this metabolic remodeling is hindered by the lack of high-depth proteomic resources and the fragmented genome annotations, which fail to capture Euglena’s proteomic diversity and plasticity. Here, we provide a comprehensive Euglena proteome landscape through advanced LC-MS/MS analysis. By leveraging the recently refined proteome annotation, we identified 9,122 protein groups, 12,027 phospho-peptides, and 3,346 ubiquitinated peptides, establishing a comprehensive Euglena’s proteome reference. Our results demonstrate that anaerobic adaptation is characterized by distinct phosphorylation and ubiquitination signatures that often occur independently of changes in global protein abundance, revealing a layer of PTM-mediated control over central metabolism during anaerobic adaptation in Euglena. This study provides a foundational resource that bridges the gap between genomic potential and functional metabolic in Euglena. By resolving the interplay between protein levels and PTM-driven regulation, our work offers a new resource for investigating environmental adaptation and provides a rigorous framework for future metabolic engineering in Euglena.

## Introduction

The photosynthetic flagellate *Euglena gracilis* is a unique evolutionary lineage characterized by a chimeric cellular organization resulting from secondary endosymbiosis.^1-3^ This species exhibits high metabolic versatility depending on environmental circumstances, including adaptive transitions across fluctuating oxygen levels and shifts between autotrophy and heterotrophy.^4-9^ Beyond its fundamental biological interest, *E. gracilis* has gained prominence as a robust industrial microalga, owing to its capacity for large-scale cultivation and its metabolic uniqueness. A key feature of this adaptability is the fermentation of the storage polysaccharide paramylon into high-value wax esters under hypoxic conditions, providing a bio-based platform for the production of biofuels and oleochemicals.^7^ ^,^ ^10-13^ Therefore, understanding the regulatory mechanisms governing these metabolic shifts is essential for both advancing evolutionary cell biology and optimizing the efficiency of algae-based bioproduction.

Pioneering genomic and proteomic investigations of *E. gracilis* provided the first comprehensive framework for understanding its complex biology.^2^ Such foundational efforts advanced proteomic studies exploring various cellular states, including responses to environmental changes^4^ ^,^ ^14-18^ and organelle-specific profiles.^19-21^ However, these studies were inherently constrained by the fragmented genomic assemblies. This has been recently improved by the achievement of a chromosome-level genome assembly,^22^ which we have further enhanced through Iso-Seq-based structural annotation to establish a deduced proteome dataset.^23^ ^,^ ^24^ By accurately defining complex gene structures, this high-fidelity reference now enables the precise mapping of peptides—a prerequisite not only for superior proteomics but also for the systematic interrogation of post-translational modifications (PTMs), such as phosphorylation and ubiquitination, which were largely inaccessible under earlier genomic frameworks. These advancements are complemented by the recent establishment of molecular genetics tools, enabling functional dissection of *E. gracilis*’s unique cellular systems.^25-27^ Consequently, the research community is now also positioned to move forward with the targeted engineering of *Euglena*’s cellular systems for enhanced industrial productivity.^28-31^

Notably, *E. gracilis* exhibits strikingly narrow shifts in the global transcriptome during major environmental transitions compared to other model eukaryotes.^2^ For instance, previous transcriptomic studies under anaerobic conditions revealed that most genes involved in paramylon degradation and wax ester fermentation show minimal changes at the mRNA level.^32^ These observations have led to the prevailing hypothesis that the primary regulatory dynamics in *E. gracilis* occur at the post-transcriptional and post-translational levels rather than through transcriptional reprogramming. This inherent transcriptional stasis necessitates a more comprehensive evaluation of the cellular response at the protein and post-translational levels. However, the extent of such proteomic regulation remains largely unquantified, prompting a central inquiry: how is the *E. gracilis* metabolic transition systematically partitioned across the global and post-translational proteomic layers? By addressing this through the application of updated structural gene annotation, we sought to determine the relative magnitude and coordination of protein abundance, phosphorylation, and ubiquitination in driving *Euglena*’s physiological response.

Here, we present a comprehensive proteomic resource for *E. gracilis* that integrates global protein abundance with site-specific phosphorylation and ubiquitination profiles during the transition from aerobiosis to anaerobiosis. By leveraging a refined deduced proteome reference, we identified 9,122 protein groups, 12,027 phospho-peptides, and 3,346 ubiquitinated peptides, providing a high-resolution data landscape of the metabolic shifts underlying paramylon and wax ester metabolism. By resolving the interplay between protein levels and PTM-mediated regulation, our work offers an extensive dataset for investigating environmental adaptation and establishes a systematic framework for metabolic engineering for biotechnological applications in *E. gracilis*.

## Materials and Methods

### Euglena strain and cell culture

*Euglena gracilis* strain NIES-48 was obtained from the Microbial Culture Collection at the National Institute for Environmental Studies (NIES), Tsukuba, Japan. Cells were maintained in citrate-free Cramer-Myers (modified CM)^33^ medium (pH 3.5) containing 0.1% (v/v) ethanol at 26 °C under continuous illumination at 50 μmol m^−2^ s^−1^ with rotary shaking at 120 rpm. After 7 days of culture, by which time the cells had reached stationary phase, cells were inoculated at an initial cell density of 5 × 10^4^ cells mL^−1^ in modified CM medium (pH 3.5) and cultured at 120 rpm and 26 °C under 50 μmol photons m^−2^ s^−1^ light (12-h:12-h light-dark cycle). For CO_2_ supplementation, a 5% CO_2_-air mixture was delivered by direct bubbling into the culture medium at a flow rate of 120 mL min^−1^. After 7 days of culture, the cells were centrifuged using a swing rotor at 2,800 × *g* for 5 min, and the medium was replaced with nitrogen-free modified CM medium (pH 3.5), and the cells were cultured under the same conditions as above for 3 days (aerobic sample). The remaining culture was transferred to a 50 mL tube, sealed, and incubated in the dark without shaking for 2 days (anaerobic sample). Cells were harvested by centrifugation as above, washed once with sterile water, and stored at −80 °C until protein extraction. Three biological replicates were prepared for each condition.

### Reference proteome from our re-annotated EugrA1 genome

The protein sequence database used in this study was derived from our previously released Iso-Seq-based re-annotation of the *Euglena gracilis* EugrA1 genome (Figshare, DOI: 10.6084/m9.figshare.c.7576343.v1)^23^ comprising 46,468 deduced protein sequences anchored to 46 chromosomes. This dataset served as the reference for all subsequent mass spectrometry searches. Functional annotation of the deduced proteins was performed by PANTHER (v19.0)^34^ using the classification scheme, with hits accepted at a threshold of *p* < 0.05. Gene Ontology (GO) terms were assigned based on the PANTHER output and used for downstream functional enrichment analysis.

### Proteome analyses

Cell pellets were lysed in 6 M guanidine-HCl containing 100 mM HEPES-NaOH, pH 7.5, 10 mM TCEP, and 40 mM chloroacetamide. Samples were heated at 95 °C, sonicated, and centrifuged, and the supernatants were collected. For global proteome analysis, 50 µg of protein was used per sample, whereas 500 µg was used for phosphoproteome and ubiquitome analyses. Proteins were purified by methanol/chloroform precipitation, resuspended in 0.1% RapiGest (Waters) in 50 mM triethylammonium bicarbonate, and digested overnight at 37 °C with Trypsin/Lys-C mix (Promega) at an enzyme-to-protein ratio of 1:100. For global proteome analysis, the resulting peptides were acidified with TFA, centrifuged, desalted using GL-Tip SDB (GL Sciences), and subjected to LC-MS/MS analysis. For phosphoproteome analysis, phosphopeptides were enriched using the High-Select Fe-NTA Phosphopeptide Enrichment Kit (Thermo Fisher Scientific) according to the manufacturer’s protocol. Enriched phosphopeptides were acidified, desalted using GL-Tip SDB, and analyzed by LC-MS/MS. For ubiquitome analysis, K-*ε*-GG-modified peptides were enriched using the PTMScan HS anti-K-*ε*-GG immunoaffinity magnetic beads (Cell Signaling Technology). Enriched peptides were eluted with 60% acetonitrile/0.1% TFA, desalted using GL-Tip SDB, and analyzed by LC-MS/MS. LC-MS/MS analysis were performed using a timsTOF HT mass spectrometer coupled to a nanoElute 2 UHPLC system (Bruker). Global proteome samples were analyzed by data-independent acquisition, and the raw data were processed using DIA-NN. Phosphoproteome and ubiquitome samples were analyzed by data-dependent acquisition, and the raw data were processed using FragPipe. Database searches were performed against the re-annotated EugrA1 protein sequence database. Trypsin specificity was used, and carbamidomethylation of cysteine was set as a fixed modification. Oxidation of methionine and protein *N*-terminal acetylation were set as variable modifications. Phosphorylation of serine, threonine, and tyrosine were additionally included for phosphoproteome analysis, and di-glycine modification of lysine was included for ubiquitome analysis. Identifications were filtered at a false discovery rate of 1%, and intensity values were used for downstream quantitative analysis.

### Differential expression analysis

The proteome datasets were processed with the DEP package (v1.32.0)^35^ in R (v4.5.2). Protein groups or peptides were retained if at least two of three biological replicates were quantified in at least one condition, normalized by variance-stabilizing transformation (VSN), and missing values were imputed under a missing-not-at-random assumption using the MinProb method (q = 0.01, set.seed(123)). Differential abundance between aerobic and anaerobic conditions was tested with test_diff (limma-based moderated t-test, with aerobic as the control), and proteins were considered significantly regulated at a Benjamini-Hochberg adjusted *p*-value < 0.05 and |log₂ fold-change| ≥ 0.58 (≥1.5-fold). Gene Ontology (GO) over-representation analysis was performed on the up- and down-regulated protein sets using the enricher function of clusterProfiler (v4.18.4)^36^ with all proteins tested for differential expression used as the background universe, and the PANTHER-based GO annotation of the EugrA1 proteome.

### Functional enrichment and motif analyses

Enrichment of kinase consensus motifs was assessed for the unique phospho-S/T sites identified in this study using a binomial test against a background of unique S/T-centered 15-residue windows extracted from the re-annotated EugrA1 proteome. Thirty-two sequence-encoded motifs from the curated library of Bradley and Beltrao^37^ were tested, and the phospho-dependent S/T-x-pS arm of the composite S/T-x-E/pS motif was additionally evaluated by cross-referencing the + 2 position of each phospho-S/T site against the empirical phospho-S map of this study. To characterize local phosphorylation clustering, the proportion of central phospho-S and phospho-T sites carrying an additional detected phosphorylation at each offset from −5 to + 5 residues was computed and stratified by the residue type (phospho-S or phospho-T) of the flanking site. *De novo* motif discovery on phospho-Y sites was performed with motif-x algorithm^38^ using rmotifx package (v1.0)^39^ using 446 unique phospho-Y 15-mers as foreground and unique Y-centered proteome windows as background (central.res = Y, min.seqs = 15, *p* < 1 × 10^−5^).

## Results

### A high-resolution proteomic landscape of *Euglena gracilis* enabled by Iso-Seq-based genomic re-annotation

To delineate the comprehensive molecular architecture governing the anaerobic response in *Euglena gracilis*, we established a multi-layered proteomic workflow integrating global protein expression with deep phosphoproteomic and ubiquitomic profiling (Figure 1A). Recognizing the inherent complexity of the *E. gracilis* genome—characterized by paralogs and unconventional gene structures—we first leveraged long-read Iso-Seq-based transcriptome datasets to refine the EugrA1 genome annotation. This process provided a high-fidelity reference of 46,468 deduced protein sequences anchored to 46 chromosomes, ensuring that our subsequent mass spectrometry-based interrogation was built upon a precise and organism-specific transcriptomic foundation (Figure 1B). Using this reference, across six biological replicates, we consistently identified approximately 8,900 protein groups, providing a broad functional classification (Figure 1C). Our global proteomic analysis captured a clear proteomic transition between aerobic and anaerobic conditions; principal component analysis (PCA) revealed a stark separation along the PC1 axis, which accounted for 57.8% of the total variance (Figure 1D). This clear segregation between the aerobic and anaerobic culture conditions suggests a global proteome-level reconfiguration under oxygen-depleted conditions. Beyond simple abundance changes in the proteome, we sought to map the regulatory landscape through post-translational modification (PTM) profiling, identifying thousands of high-confidence phosphorylation and ubiquitination (K-GG) sites. The phosphoproteome profile exhibited a characteristic distribution of Ser (72.4%), Thr (23.8%), and Tyr (3.8%), with a substantial fraction of the proteome harboring multiple modification sites (Figure 1E). Similarly, the ubiquitome analysis identified thousands of K-GG sites, with over 700 protein groups being modified at a single site and several hundred protein groups showing polyubiquitination or multiple ubiquitination sites (Figure 1F). Collectively, this dataset integrates Iso-Seq-based genomic refinement with deep, multi-layered proteomic profiling, providing a robust informational resource to explore both the expression-level responses and the intricate post-translational regulation mediating the transition between aerobic and anaerobic life in *E. gracilis*.

**Figure 1.**
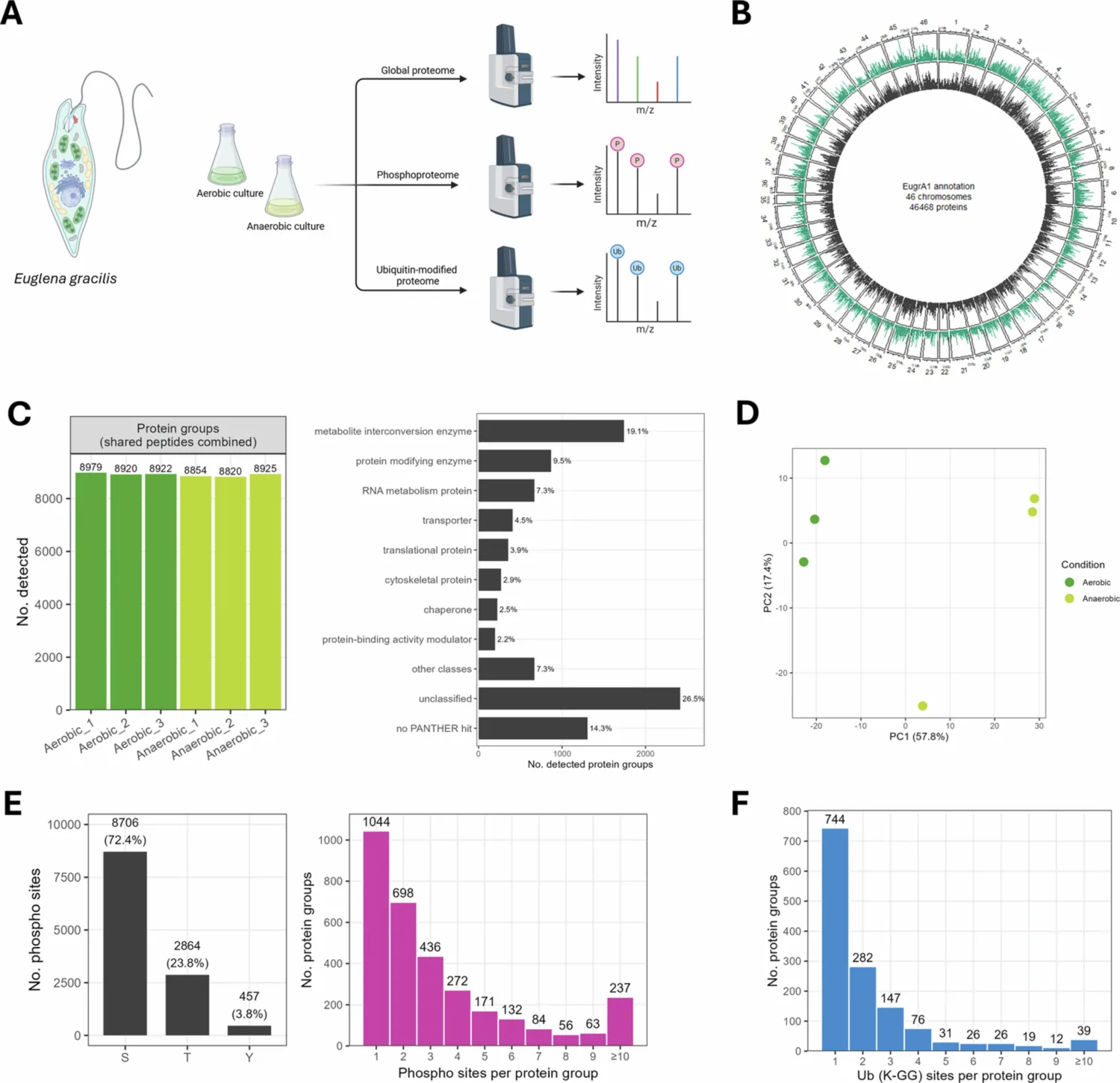
**A high-resolution multi-proteomic landscape of** ***Euglena gracilis*** **enabled by Iso-Seq-based genomic re-annotation. (A)** Experimental workflow for the integrated multi-omics analysis. *E. gracilis* cells were cultured under aerobic and anaerobic conditions, followed by LC-MS/MS-based profiling of the global proteome, phosphoproteome, and ubiquitome (Created in BioRender). **(B)** Circos plot illustrating the refined EugrA1 genome annotation incorporating Iso-Seq datasets. The tracks display the 46 chromosomes, the distribution of total annotated gene models (grey), and the genomic loci of proteins identified in the global proteome (teal). **(C)** Identification of protein groups and functional coverage. (Left) Number of protein groups detected across six biological replicates (*n* = 3 per condition). (Right) Functional classification of detected proteins (black) against total annotated proteins (grey) based on PANTHER categories, with percentage coverage indicated. **(D)** Principal component analysis (PCA) of the global proteome (*n* = 3 per group) across aerobic and anaerobic conditions. **(E)** Characterization of the phosphoproteome. (Left) Distribution of phosphorylated Serine (S), Threonine (T), and Tyrosine (Y) residues. (Right) Number of identified phosphorylation sites per protein. **(F)** Distribution of ubiquitination (K-GG) sites identified per protein.

### Differential proteomic profiling between aerobic and anaerobic conditions

To identify proteins with significant abundance changes between the two conditions, we performed a differential expression analysis, visualized via a volcano plot (Figure 2A). Through the GO enrichment analysis based on the updated EugrA1 annotation, we were able to precisely delineate the functional consequences of these proteomic shifts (Figure 2B). Under aerobic conditions, *E. gracilis* prioritized energy-intensive processes, as evidenced by the significant enrichment of GO terms related to active transport (P-type ATPase-coupled) (GO:0042626) and proton transmembrane transport (GO:1902600). Additionally, lytic processes involving cysteine proteases (GO:0005764; GO:0005773; GO:0004197; GO:0008234) and plasma membrane (GO:0005886) activities were prominent, suggesting active cellular turnover and environmental interaction. In contrast, anaerobic adaptation triggered a specialized metabolic shift. We observed a distinct enrichment of terms associated with mitochondrion activity (GO:0005739) and succinate/fumarate fermentation (GO:0043648), consistent with the activation of the wax ester fermentation pathway. The upregulation of NAD(P)H-dependent oxidoreductase activities further underscores the redox balancing required under oxygen-limited conditions. Furthermore, “negative regulation of signaling” and “response to stimulus” were enriched in the anaerobic-upregulated proteome, indicating a coordinated suppression of certain cellular pathways to conserve energy during the transition to an anaerobic state. Collectively, these results demonstrate that *E. gracilis* undergoes a dynamic cellular reconfiguration in response to the anaerobic conditions, which may facilitate a prioritized shift toward specialized anaerobic fermentation pathways.

**Figure 2.**
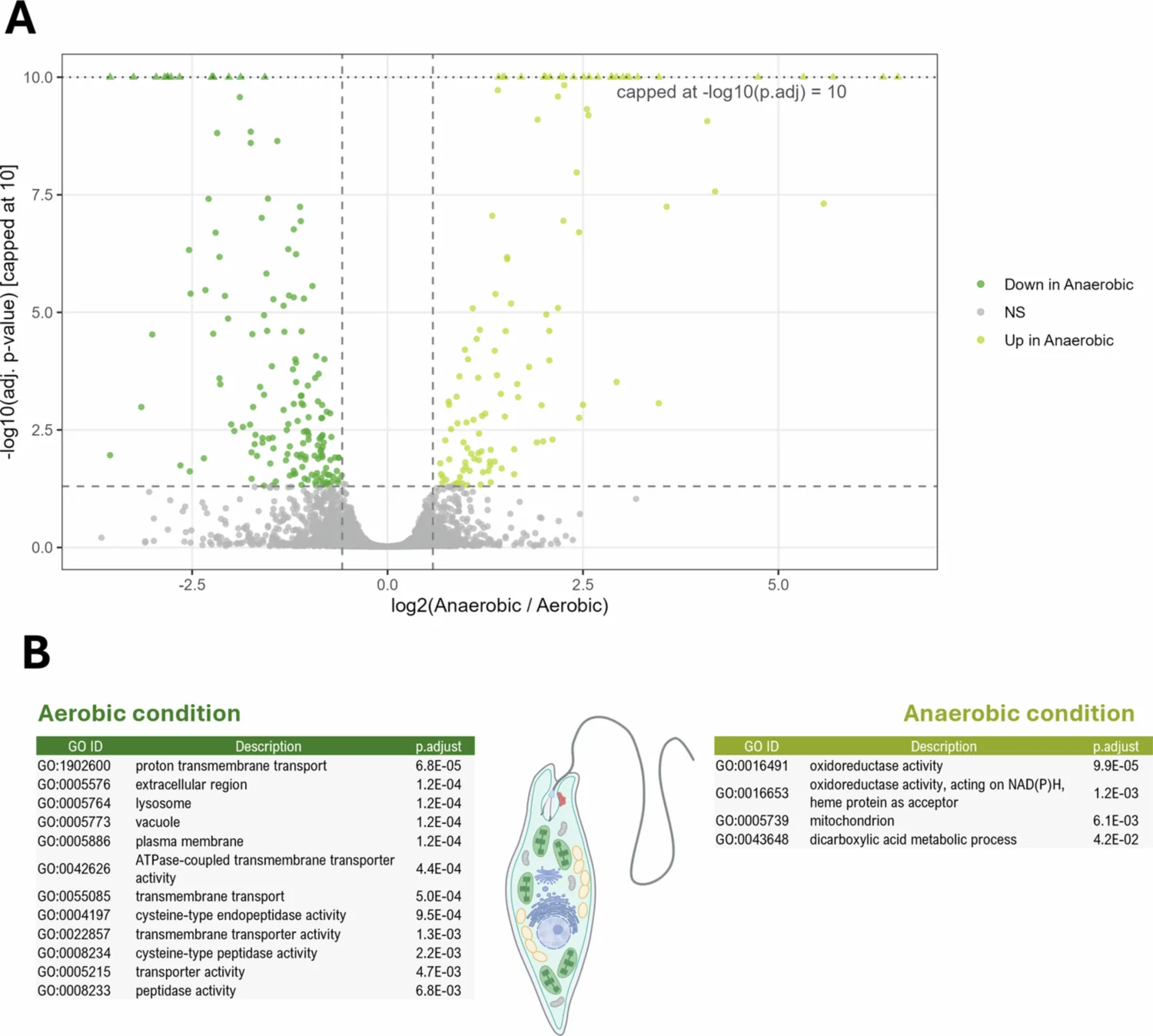
**Differential proteomic and functional landscape of** ***E. gracilis*** **under anaerobic transition. (A)** Volcano plot showing the differentially expressed proteins between aerobic and anaerobic conditions. The log_2_ fold change (Anaerobic/Aerobic) is plotted against the -log_10_ adjusted *p*-value. Significant proteins were defined by a threshold of False Discovery Rate (FDR) < 0.05 and an absolute log_2_ fold change > 0.58 (representing a 1.5-fold change). Proteins significantly up-regulated in anaerobic conditions are shown in yellow-green, while those down-regulated are in green. For improved visibility, *p*-values exceeding the range are capped at -log_10_(p.adj) = 10. **(B)** Functional enrichment analysis of differentially expressed proteins (Created in BioRender). GO enrichment analysis based on the EugrA1 annotation identified GO terms enriched under aerobic (left, green) and anaerobic (right, yellow-green) conditions. The corresponding adjusted *P* values are shown.

### Regulatory motif exploration of the *E. gracilis* phosphoproteome

To delineate the kinase signatures in the *E. gracilis* phosphoproteome, we assessed the 11,219 unique phospho-S/T sites for enrichment of 33 consensus motifs derived from Bradley and Beltrao,^37^ the S/T phosphorylation motif set against an unmodified proteome background (Figure 3A). Twenty-six of the 32 sequence-encoded motifs reached significance, dominated by the proline-directed S/T-P (*n* = 3,661, -log_10_(*p*-value) > 300) and the basophilic R-x-x-S/T (*n* = 1,815, -log_10_(*p*-value) > 219.9), associated with the activities of MAPK/CDK and CDPK/CaMK families, respectively. Stricter compound motifs such as P-x-S/T-P (MAPK), S/T-P-R/K and R-x-x-S/T-P, were likewise enriched, while the acidophilic CK2 signatures (S/T-x-x-D/E; S/T-x-E) and the ATM/ATR-associated S/T-Q motif appeared at more modest but significant levels, collectively indicating that *E. gracilis* retains a canonical complement of eukaryotic kinase activities despite its evolutionary divergence. Strikingly, the phospho-dependent S/T-x-pS motif, evaluated against the empirical phospho-S map based on this study, showed a strongly enriched signature of the panel (*n* = 960), suggesting clustered phosphorylation at the + 2 position. To resolve this clustering more comprehensively, we computed the proportion of phospho-S (*n* = 8,706) and phospho-T (*n* = 2,864) sites carrying an additional phosphorylation at each offset from −5 to + 5 residues, stratified by whether the flanking site was a phospho-S or a phospho-T (Figure 3B). Pronounced peaks at the ± 1 and ± 2 positions demonstrate that co-modified residues preferentially cluster within a ± 2-residue window of the central site, consistent with processive or priming-dependent kinase action. We further interrogated tyrosine phosphorylation by *de novo* motif-x-based discovery on the 446 unique phospho-Y sequences (Figure 3C). Nine motifs reached significance and fell into two distinct classes: proline-coupled signatures with proline downstream of the phospho-tyrosine (Y-S-P, motifx score 3.17 × 10^2^; P-x-Y-x-P, 1.58 × 10; Y-x-x-P, 5.98), reminiscent of MAPK- or CMGC-family consensus arrangements rather than classical receptor-tyrosine-kinase substrates, and a set of motifs featuring serine or threonine residues within ± 2 of the central tyrosine (Y-S, Y-P-Y, Y-P, Y-x-x-S, S-Y), indicating that phospho-Y sites in *E. gracilis* are preferentially embedded in S/T-rich sequence neighborhoods. Together, these analyses establish that phosphorylation in *E. gracilis* is governed by a canonical yet diversified set of kinase consensus motifs. Furthermore, we examined condition-specific regulatory protein phosphorylation under anaerobiosis to identify responsive pathways. Phosphosites that were significantly decreased under anaerobic conditions showed a notable enrichment for terms related to the cell periphery (GO:0071944) and plasma membrane (GO:0005886). Conversely, anaerobic-upregulated phosphorylation was enriched on proteins associated with nuclear processes, including chromosome and chromatin organization (Figure 3D). We also asked whether phosphorylation of MAP kinase-related proteins shows condition-specific patterns, by exploring proteins annotated with MAP kinase-related conserved domains (Supplementary Table S1-S2). Among 11,219 identified phosphosites, 150 mapped to 36 MAP kinase-related protein groups spanning 15 annotated kinase types (Figure 3E), of which 8 were phosphorylated exclusively under either aerobic or anaerobic conditions (Figure 3F). Collectively, these data provide a foundational dataset illustrating the distribution and intensity of site-specific phosphorylation underlying the broader cellular reconfiguration in the anaerobic metabolic states in *E. gracilis*.

**Figure 3.**
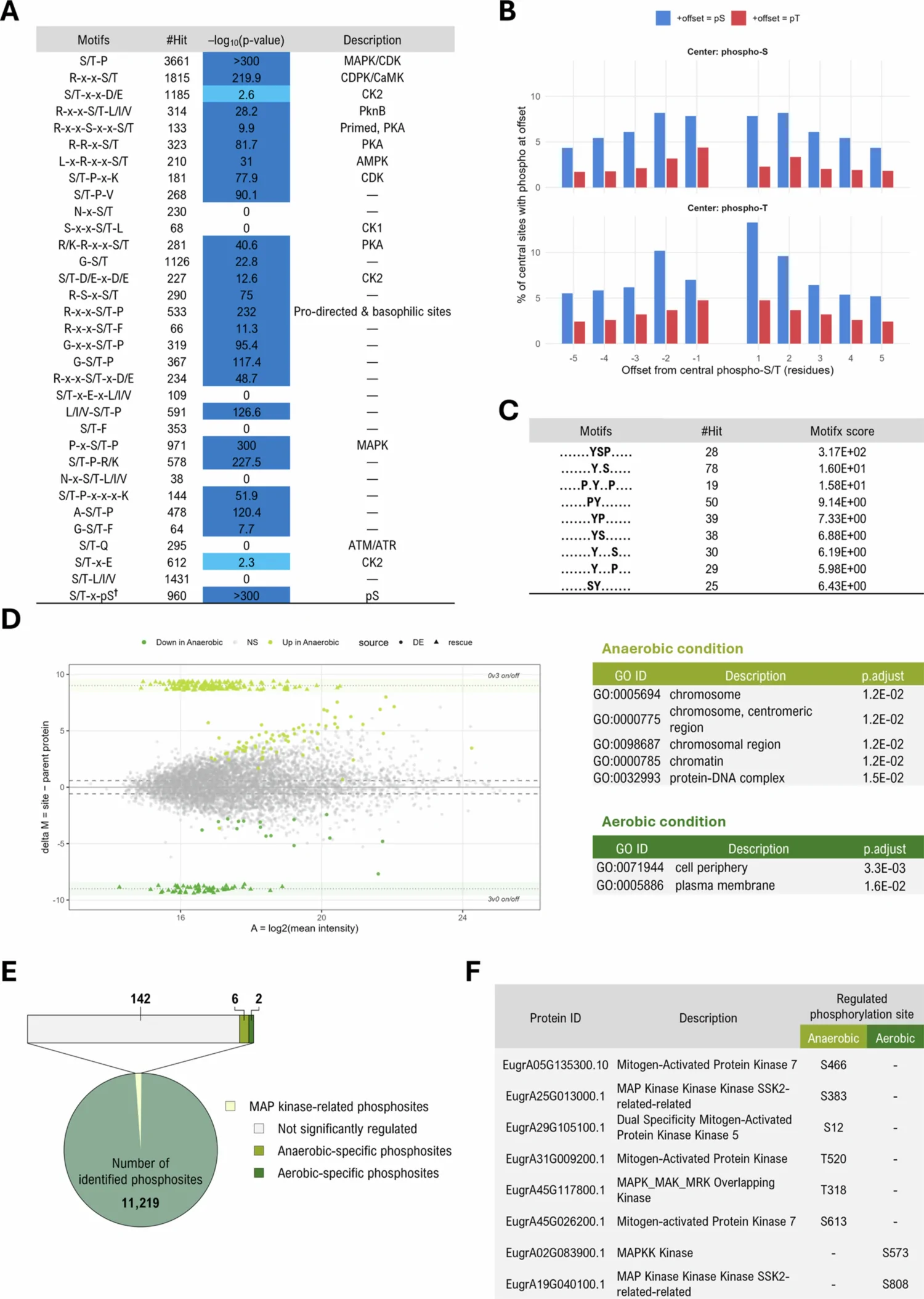
**Phosphoproteome under anaerobic and aerobic conditions in** ***E. gracilis***. (**A**) Enrichment of 33 phosphorylation consensus motifs in 11,219 unique phospho-S/T sites. Each motif was tested by binomial test against the background of 977,923 unique S/T 15-mer windows derived from the annotated protein set. The table reports the number of foreground hits (#Hit) and -log_10_(*p*-value); cells are colored by significance (dark blue, *p* < 10^−6^; cyan, 10^−4^ < *p* < 10^−2^; white, *p* > 10^−2^). The right-most column indicates the canonically related kinase family or family-level annotation. Thirty-two motifs are derived from the Bradley and Beltrao^37^ library (with the composite S/T-x-E/pS decomposed into its E component, S/T-x-E); the bottom row (S/T-x-pS^†^) corresponds to the pS component, evaluated by cross-referencing the + 2 position of each phospho-S/T site against the detected phospho-S map data in this study. †For this pS component, the motif was tested against a background of unique S/T 15-mer windows derived from annotated proteins with a detected phosphorylation site (*n* = 190,715), rather than the whole-proteome background used for the other 32 motifs. **(B)** Distance-dependent cluster phosphorylation profile. Percentage of central phosphosites with a detected phospho-S (blue) or phospho-T (red) at offsets ± 1 to ± 5 from the centered phosphosite. Top: phospho-S sites (*n* = 8,706); bottom: phospho-T sites (*n* = 2,864). **(C)** *De novo* motif-x-based discovery on phospho-Y sites. Motif-x was applied to 446 unique phospho-Y 15-mer sequences using 175,275 unique Y-centered proteome windows as background (central.res = Y, min.seqs = 15, *p*-value < 10^−5^). **(D)** Comparative analysis of site-specific phosphorylation dynamics. MA-plot represents the differential abundance of unique phosphorylation sites between aerobic and anaerobic conditions. The Y-axis (Delta M) represents the log_2_ fold change of phosphosite intensity. Additionally, phosphosites characterized by their presence or absence in a condition-specific manner are indicated at the top (0v3: anaerobic-specific) and bottom (3v0: aerobic-specific) of the plot, labeled as “rescue” (triangles). GO enrichment analysis of differentially regulated phosphosites. (Top) GO terms enriched in anaerobic-upregulated (yellow-green) sites. (Bottom) GO terms enriched in aerobic-upregulated (green) sites. The tables list the GO ID, description, and adjusted *p*-values (p.adjust) < 0.05. **(E)** MAP kinase-related phosphosites (142 non-significant, 6 anaerobic-specific, 2 aerobic-specific). Detailed MAP kinase-related functional annotations and phosphosites are provided in Supplementary Table S1 and Supplementary Table S2, respectively. **(F)** Proteins carrying the anaerobic- and aerobic-specific phosphosites shown in (E).

### Proteasome ubiquitination sites in response to anaerobic transition

Moreover, we simultaneously characterized the ubiquitome of *E. gracilis* to identify condition-specific degradation signaling. The differential analysis of K-GG sites revealed that the majority of significant changes were characterized by their presence or absence in either aerobic or anaerobic conditions (Figure 4A). Through the GO enrichment analysis of these differentially regulated sites, we found a striking concentration of terms related to the proteasome complex (GO:0000502) and its associated regulatory units (Figure 4A). Given the critical role of the proteasome, we cross-referenced these findings with our global proteome dataset. A Venn diagram illustrated that these three proteins showing increased ubiquitination under anaerobic conditions were among the most abundant proteins within the “proteasome” functional category (Figure 4B). Specifically, the subunit encoded by EugrA09G057700.1 was identified as the most abundant proteasome-related protein in the entire proteome (534/15116), with other subunits also ranking within the top 20% of the most abundant proteins in the proteome (EugrA46G036200.1: 1169/15116, EugrA32G069400.1: 2726/15116). However, while their abundance levels remained high and stable, showing no significant changes between aerobic and anaerobic conditions (Figure 4C), their respective ubiquitination sites exhibited dynamic, condition-specific shifts (Figure 4D). These findings suggest that the targeted ubiquitination of the proteasome machinery represents a key regulatory hallmark characterizing the cellular state of *E. gracilis* under anaerobic conditions.

**Figure 4.**
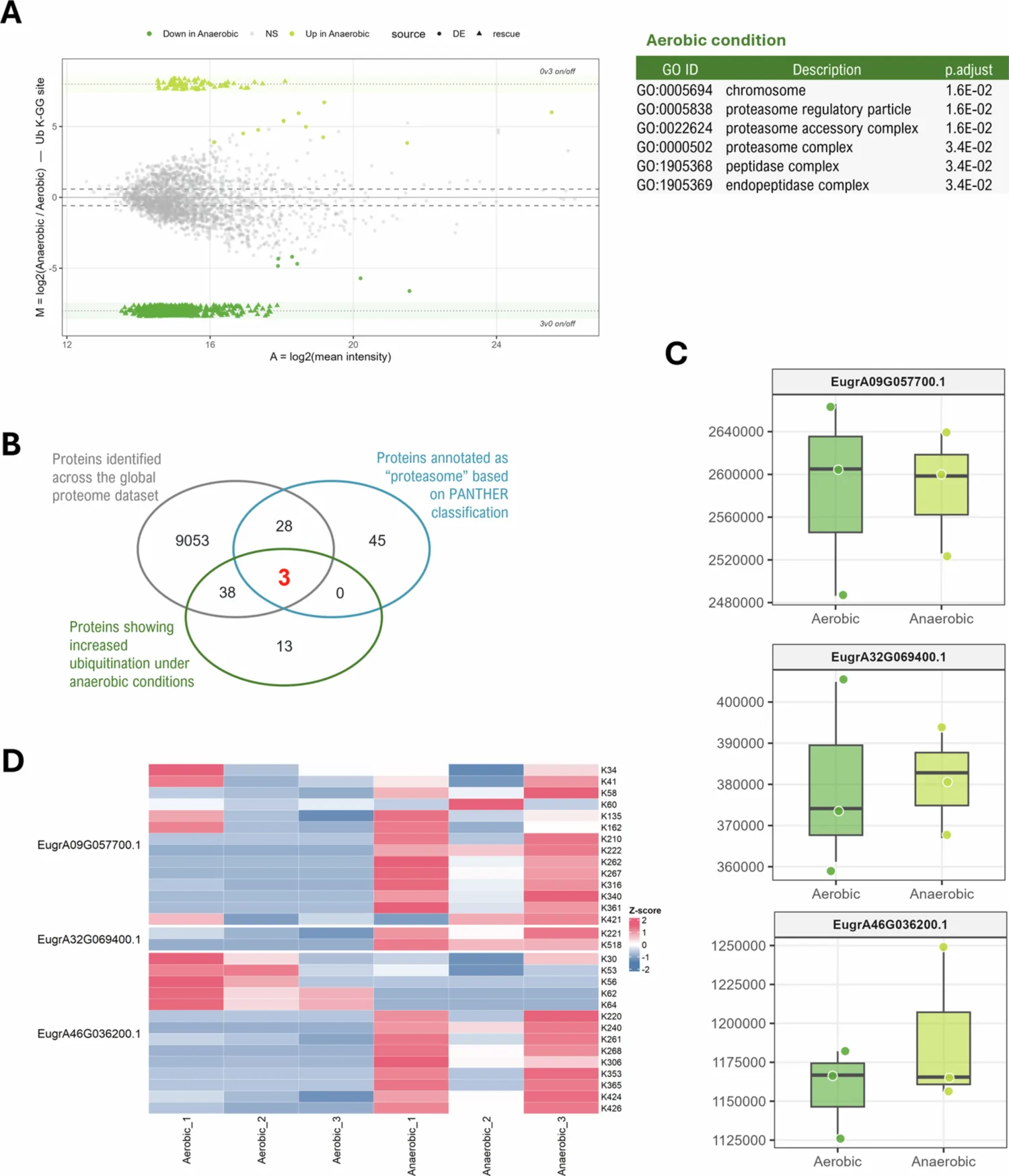
**Ubiquitome dynamics and targeted ubiquitination of the proteasome under anaerobic conditions.** (**A**) Comparative analysis of site-specific ubiquitination dynamics. MA-plot represents the differential abundance of ubiquitination (K-GG) sites between aerobic and anaerobic conditions. The Y-axis (M) represents the log2 fold change of ubiquitination site intensity. Additionally, ubiquitination sites characterized by their presence or absence in a condition-specific manner are indicated at the top (0v3: anaerobic-specific) and bottom (3v0: aerobic-specific) of the plot, labeled as “rescue” (triangles). GO enrichment analysis of anaerobic-upregulated ubiquitination sites, highlighting the significant enrichment of the proteasome complex and peptidase activities. (**B**) Venn diagram cross-referencing the global proteome, proteins annotated as “proteasome” (PANTHER), and proteins with increased ubiquitination. The three overlapping proteins are highly abundant across the global dataset. (**C**) Boxplots of protein-level abundance for the corresponding putative proteasome subunits. (**D**) Heatmap of Z-scored intensities for specific K-GG sites on each of the three putative proteasome subunits.

### Multi-layered differential regulation of enzymes involved in paramylon degradation and wax ester fermentation under anaerobic conditions

By mapping our proteome abundance, phosphoproteome, and ubiquitome datasets onto enzymes known to be involved in the paramylon degradation and wax ester fermentation pathway in *E. gracilis*, we assessed their differential regulation under aerobic and anaerobic conditions (Figure 5, Supplementary Table S3-S6). We found that proteome abundance was differentially regulated between the two branches, with glucan synthase-like 1 (GSL1) significantly reduced, whereas phosphoenolpyruvate carboxykinase (PEPCK), NADP + -dependent malic enzyme (ME1), pyruvate:NADP + oxidoreductase (PNO), propionyl-CoA carboxylase (PCC), and wax ester synthase (WSD2) were markedly upregulated. We also observed that, under anaerobic conditions, PNO and electron transfer flavoprotein (ETF) underwent condition-specific phosphorylation, while glucan synthase-like 2 (GSL2) was dephosphorylated. We further identified ubiquitination on GSL1, GSL2, PEPCK, the paramylon-degrading *β*-1,3-glucan phosphorylase (P1), and *trans*-2-enol-CoA hydratase (ECH), although none of these sites differed significantly between aerobic and anaerobic conditions. Together, these datasets provide a multi-layered view of enzyme regulation, spanning protein abundance and phosphorylation, across the paramylon degradation and wax ester fermentation pathway in *E. gracilis*, representing modification-specific regulation as well as coordinated regulation across protein abundance and modification.

**Figure 5.**
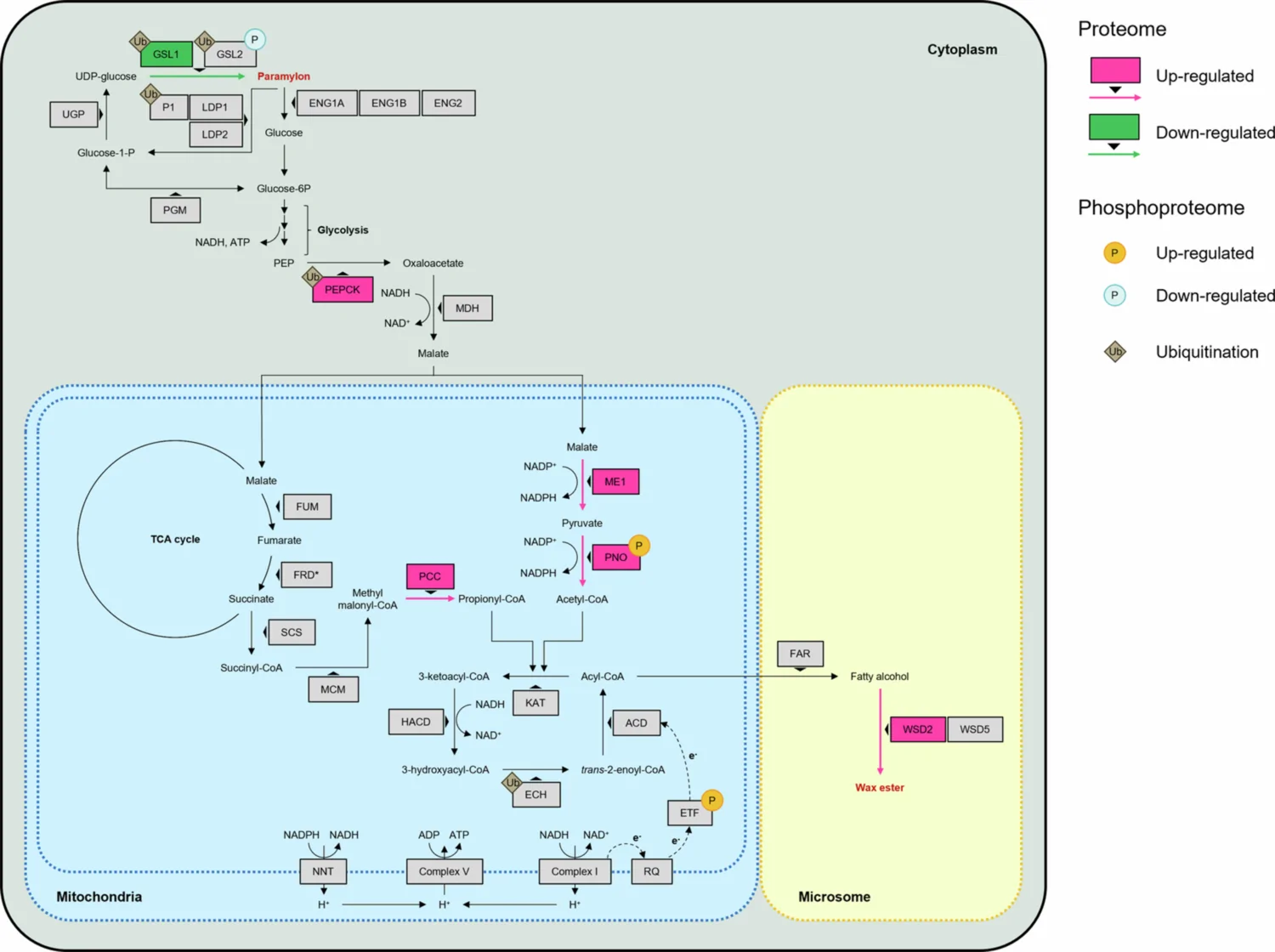
**Schematic representation of paramylon degradation and the wax ester fermentation pathway under anaerobic conditions.** Differentially expressed proteins (|log_2_ fold change| > 0.58, p.adj < 0.05) are highlighted in green (upregulated) and red (downregulated) boxes. Upregulated and downregulated reactions are indicated by green and red arrows, respectively. Grey boxes and black arrows denote proteins and reactions that remain unchanged under anaerobic conditions, respectively. The phosphorylation status is indicated by ‘P’ circles (orange, upregulated; light blue, downregulated) and identified ubiquitination sites are highlighted with brown diamonds. PEP, phosphoenolpyruvate; GSL, glucan synthase-like; UGP, UDP-glucose pyrophosphorylase; ENG, *β*-1,3-endo-glucanase; LDP, laminaridextrin phosphorylase; P1, *β*-1,3-glucan phosphorylase; PGM, phosphoglucomutase; PEPCK, phosphoenolpyruvate carboxykinase; MDH, malate dehydrogenase; ME1, NADP + -dependent malic enzyme; PNO, pyruvate:NADP + oxidoreductase; FUM, fumarate hydratase; FRD, fumarate reductase; SCS, succinyl-CoA synthetase; MCM, methylmalonyl-CoA mutase; PCC, propionyl-CoA carboxylase; KAT, 3-ketoacyl-CoA thiolase; HACD, 3-hydroxyacyl-CoA dehydrogenase; ECH, *trans*-2-enol-CoA hydratase; ACD, acyl-CoA dehydrogenase; NNT, nicotinamide nucleotide transhydrogenase; ETF, electron transfer flavoprotein; FAR, fatty acyl-CoA reductase; WSD, wax ester synthase/diacylglycerol acyltransferase (WS/DGAT). *The FRD was identified in the deduced protein references but not detected in the proteome datasets. The schematic pathway is adapted from Nakazawa and Inui,^40^ Tamaki et al.^18^, Padermshoke et al.^41^, and Yoshida et al.^32^. The protein query ID and corresponding identified protein ID in the re-annotated EugrA1 database are described in Supplementary Table S3. Detailed protein abundance, phosphorylation, and ubiquitination are provided in Supplementary Table S4-S6.

## Discussion

This study presents the first integrated multi-layered proteomic resource for *E. gracilis*, combining global protein abundance with site-resolved phosphorylation and lysine ubiquitination, each profiled in parallel under aerobic and anaerobic conditions (Figure 1A). To our knowledge, it is the first proteomic interrogation of *E. gracilis* conducted against a chromosome-level annotated gene structure: by anchoring the analysis to an Iso-Seq-refined re-annotation of the EugrA1 genome (Figure 1B), we obtained the genomic reference required for accurate spectral assignment in a genome complicated by paralogs and unconventional gene structures. Across six samples we consistently detected ~8,900 protein groups (Figure 1C, D) and tens of thousands of high-confidence modification sites (Figure 1E, F), establishing an *E. gracilis* genomic resource for system-level interrogation.

The Iso-Seq-based re-annotation provided full-length transcript models and thereby precise coordinates for each modification site. This allowed us to delineate, to the best of our knowledge, the first proteome-wide phosphorylation landscape reported for *E. gracilis*—and indeed for any euglenids—encompassing 12,027 high-confidence sites and a canonical complement of eukaryotic kinase consensus motifs: proline-directed (MAPK/CDK), basophilic (CDPK/CaMK, PKA, AMPK), acidophilic (CK2) and DDR-associated (ATM/ATR) (Figure 3A). We could also trace a canonical MAP kinase phosphorylation cascade in *E. gracilis*, including some condition-specific regulation between aerobic and anaerobic states (Figure 3E, F). The retention of such a canonical motif repertoire echoes observations in large-scale phosphoproteomes of related kinetoplastid parasites,^42-44^ suggesting broad conservation of phosphosignalling architecture across the Excavata. We further observed that a substantial fraction of phosphosites cluster within ± 2 residues of one another—a pattern consistent with the broadly conserved tendency, documented in budding yeast CDK substrates,^45^ animals^46^ and *Arabidopsis,* ^47^ for proximal phospho-residues to act as cooperative or primed regulatory units rather than as isolated events. The strong enrichment of the prominent + 1 peak for phospho-T-centered sites may suggest that combinatorial phospho-coding through priming-dependent processive kinase action may contribute to signal integration in *E. gracilis* (Figure 3B). Superimposed on this canonical architecture, the aerobic-to-anaerobic transition was accompanied by a coherent spatial reconfiguration of the phospho-landscape, in which membrane-associated phosphorylation was preferentially attenuated while nuclear and chromatin-associated phosphorylation was selectively induced (Figure 3D)—a pattern that mirrors the global proteome-level shifts (Figure 2) and may transition toward distinct regulatory regimes governing different cellular compartments and metabolism under anaerobic conditions.

The ubiquitination profile reported here, to our knowledge, likewise constitutes the first proteome-wide ubiquitome described for *E. gracilis* and for the Euglenida. A notable feature of this ubiquitome was the condition-specific ubiquitination of multiple proteasome subunits despite essentially unchanged subunit abundance, indicating that the proteasome itself is subject to post-translational modification during the anaerobic transition (Figure 4). Regulatory ubiquitination of proteasome subunits has been documented across diverse eukaryotes as a context of modulating proteasome activity and turnover: in budding yeast, monoubiquitination of the ubiquitin-receptor subunit Rpn10 by the Rsp5 ligase regulates substrate recruitment to the proteasome^48^; in mammalian cells, autoubiquitination of Rpn13 by the proteasome-associated ligase Ube3c/Hul5 attenuates the proteasome's capacity to bind and degrade ubiquitin conjugates^49^; and in *Arabidopsis*, extensive ubiquitination of 26S subunits marks inactive proteasomes for selective autophagic clearance via the RPN10 receptor.^50^ The patterns observed in *E. gracilis* suggest that condition-specific ubiquitination of the proteasome might be involved in its anaerobic response, although the functional consequences of such modifications identified here remain elusive.

Mapping these datasets onto the paramylon-degradation and wax-ester-fermentation pathway (Figure 5) places the anaerobic response in its metabolic context. The suppression of GSL1 alongside the activation of the fermentation branch is consistent with the known redirection of carbon flux from paramylon storage toward wax ester biosynthesis during the aerobic-to-anaerobic transition. We found that ME1 was upregulated, a change also consistent with a report that its silencing decreases wax ester production under anaerobic conditions.^40^ We also observed that PNO became more abundant and more phosphorylated under anaerobic conditions, implying that this modification may regulate its role in converting pyruvate to acetyl-CoA. We also found that PCC—involved in propionyl-CoA biosynthesis, the C3-donor for wax ester fermentation^41^—was upregulated, which might reflect concerted regulation during wax ester fermentation. Among the enzymes related to the paramylon-degradation and wax-ester-fermentation pathway, we did not observe a significant difference in ubiquitination between the conditions, whereas we found that several phosphorylation sites varied in step with the abundance of the corresponding proteins. Such coordination between phosphorylation and abundance may function to tune flux through the pathway in response to anaerobic conditions, although the functional significance of these modifications remains elusive. The availability of genome-editing approaches in *E. gracilis* ^25-27^ should also make the modification sites cataloged here amenable to targeted functional analysis, for example through substitution of individual phospho- or ubiquitin-acceptor residues, or through perturbation of candidate kinases and ligases.

## Supplementary Material

Supplementary Table S3.xlsx Supplementary Table S3.xlsx

Supplementary Table S1.xlsx Supplementary Table S1.xlsx

Supplementary Table S4.xlsx Supplementary Table S4.xlsx

Supplementary Table S5.xlsx Supplementary Table S5.xlsx

Supplementary Table S6.xlsx Supplementary Table S6.xlsx

Supplementary material table caption Supplementary material table caption

Supplementary Table S2.xlsx Supplementary Table S2.xlsx

## Data Availability

The mass spectrometry proteomics data have been deposited to the jPOSTrepo^51^ under accession codes JPST004708 (jPOSTrepo) and PXD079789 (ProteomeXchange).
