# Supplementary material for: Mapping the proteome landscape in Euglena gracilis: global, phospho, and ubiquitin proteomes under anaerobic response
Source: Plant Signal Behav. 2026 Oct 8;21(1):2720310. doi: 10.1080/15592324.2026.2720310 (PMC13660438; doi:10.1080/15592324.2026.2720310)
Supplement: Supplementary file 1 [file KPSB_A_2720310_SM7354.docx]

**Supplementary Table captions**

**Supplementary Table S1.** Putative MAP kinase-related proteins in the re-annotated EugrA1 database, based on PANTHER functional annotation.

**Supplementary Table S2.** Detection of the MAP kinase-related proteins listed in Supplementary Table S1 in the phosphoproteome dataset.

**Supplementary Table S3.** Cross-referenced mapping of genes encoding putative enzymes related to paramylon and wax ester metabolism in the re-annotated EugrA1 database.

**Supplementary Table S4.** Detection of the enzymes listed in Supplementary Table S3 in the global proteome dataset.

**Supplementary Table S5.** Detection of the enzymes listed in Supplementary Table S3 in the phosphoproteome dataset.

**Supplementary Table S6.** Detection of the enzymes listed in Supplementary Table S3 in the ubiquitome dataset.
